# Chronically administered *Agave americana var*. marginata extract ameliorates diabetes mellitus, associated behavioral comorbidities and biochemical parameters in alloxan-induced diabetic rats

**DOI:** 10.1016/j.jsps.2022.06.003

**Published:** 2022-06-13

**Authors:** Ambreen Aleem, Shahla Shahnaz, Sana Javaid, Waseem Ashraf, Muhammad Fawad Rasool, Tanveer Ahmad, Abdullah F.Alotaibi, Khalid S. Albeshri, Faleh Alqahtani, Imran Imran

**Affiliations:** a Department of Pharmacology, Faculty of Pharmacy, Bahauddin Zakariya University, 60800 Multan, Pakistan; b Department of Pharmacy, The Women University, Multan, Pakistan; c Department of Pharmacy Practice, Faculty of Pharmacy, Bahauddin Zakariya University, 60800 Multan, Pakistan; d Institute for Advanced Biosciences (IAB), Centre National de la Recherche Scientifique, Unite Mixte de Recherche 5309, Institut National de la Sante et de la Recherche Medicale U1209, Grenoble Alpes University, 38700 La Tronche, France; e Department of Pharmacology and Toxicology, College of Pharmacy, King Saud University, Riyadh 11451, Saudi Arabia

**Keywords:** Agave marginate, Alloxan-induced diabetes, Antioxidant, Open field test, Morris water maze test, Lipid profile, Polyphenols

## Abstract

**Introduction:**

Diabetes mellitus causes hyperglycemia and associated complications to the brain. In current study, the traditionally reported remedial claims of *Agave americana* var. *marginata* has been scientifically investigated in diabetic rats.

**Methodology:**

The methanolic extract of leaves of Agave *americana* var. *marginata* (Aa.Cr) was characterized for total phenols, flavonoids, and antioxidant potential through *in-vitro* testing. The rats chronically pre-treated with Aa.Cr (400 and 600 mg/kg) for 45 days were challenged with alloxan-induced hyperglycemia. The dose-dependent effects of Aa.Cr on blood glucose levels and body weights were compared with diabetic rats using glibenclamide (0.6 mg/kg) as a standard. The animals were tested for diabetes-associated neurological comorbidities through behavioral and biochemical evaluation.

**Results:**

The phenols and flavonoids enriched Aa.Cr caused a significant dose-dependent hypoglycemic effect. Aa.Cr showed protection from comorbid anxiety, depression and cognitive impairment as compared to diabetic rats. The alanine aminotransferase, total cholesterol, triglycerides and low-density lipoprotein were prominently reduced, and high-density lipoprotein was increased in rats treated with Aa.Cr. Moreover, the oxidative stress in isolated brains was reduced by Aa.Cr.

**Conclusion:**

These findings suggest that Aa.Cr is enriched with antioxidant and anti-inflammatory phytoconstituents valuable for diabetes and related neurological complications.

## 1 Introduction

Diabetes mellitus (DM) is a metabolic disorder causing elevated levels of blood glucose, due to insulin deficiency or insulin resistance. The prevalence of DM is rising exponentially across the globe, as an increase from 108 million to 425 million cases has been documented until 2017. It is important to focus on financial, social, and health systems to cope with diabetes and related comorbidities, as the statistics of diabetes would boost up to 642 million by the year 2045 (Cho et al., 2018, Glovaci et al., 2019).

Previous literature suggests that cognitive dysfunction and dementia are among the most prevailing comorbidities of DM (Jayaraj et al., 2020). Among different factors precipitating the DM-related cognitive impairment, the inadequate absorption of glucose in neurons and impaired insulin signaling are some that result in compromised energy production and brain atrophy. Other pathophysiological factors that might contribute to DM-associated cognitive decrements are oxidative stress, inflammation, the glycogen synthase kinase 3β (GSK3β) signaling mechanism, advanced glycation end-products (AGEs), protein misfolding and Aβ accumulation in the brain (Kong et al., 2020, Pugazhenthi et al., 2017). Furthermore, other concomitant psychological problems like, e.g. anxiety and depression also notably intensify the pressing problems in the daily lives of diabetics (Purewal and Fisher, 2018, Whitworth et al., 2016).

DM is treated by employing a range of glucose-lowering drugs (alone or in combination). But unfortunately, these therapeutic strategies have the possibilities to induce hypoglycemia and insulin insensitivity (Ruscica et al., 2017). Additionally, thiazolidinediones (TZDs) exert adverse effects on bone metabolism (Gilbert and Pratley, 2015). Presently, metformin is among the most commonly used biguanides, and the adverse effects include vitamin B12 insufficiency, hemolytic anemia, digestive disorders and metabolic acidosis, and hyperlactatemia occurs during beneficial dosing and coexisting conditions such as renal failure, mainly via metformin accumulation (Corchia et al., 2020, Wang and Hoyte, 2019). Traditionally, plant-based remedies have been used to alleviate a range of health disorders. Plants with antidiabetic activity can revamp glycemic control by synchronizing diabetes-associated complications (Choudhury et al., 2018, Zhang et al., 2015).

Different chemical-induced diabetes models, like, e.g. streptozotocin (nitrosourea antineoplastic agent) and alloxan-induced diabetes, have been implicated by researchers to examine the effectiveness of test treatments. Alloxan has been broadly employed by researchers to induce experimental diabetes in rodents, as it inhibits glucose-stimulated insulin secretion and causes necrosis of insulin producing cells of the pancreas (Ighodaro et al., 2017).

*Agave americana* var. *marginata,* an edible plant from the family Agavaceae, is locally known as “rail kathali” and is commonly found in South Punjab, Pakistan. The sap of *Agave americana* has been traditionally used for diuretic, laxative, immunomodulatory, anti-inflammatory and anti-hypertensive purposes (Kadam et al., 2012, Nava-Cruza et al., 2015). Its secondary metabolites (polyphenols i.e. quercetin, ellagic acid, kaempferol) possess antidiabetic (Sahnoun et al., 2019), anticancer (Anajwala et al., 2010), anti-inflammatory (Misra et al., 2018), antioxidant and antimicrobial characteristics. Previous phytochemical studies showed the presence of 141 steroidal saponins, sapogenins, flavonoids, sterols, phenolic acids, volatile coumarins, alcohols, fatty acids and agavosides (Sidana et al., 2016).

In the present study, the crude methanolic extract of *Agave americana* var. *marginata* (Aa.Cr) has been investigated to validate its claimed traditional remedial uses. The Sprague-Dawley rats were chronically treated with Aa.Cr followed by induction of alloxan-induced diabetes. The antidiabetic effects of Aa.Cr were estimated by monitoring the effects on animal’s hyperglycemia, hyperlipidemia and liver functionality parameters. The neuromodulatory potential of Aa.Cr were tested by evaluating the diabetes-associated anxiety, depression and cognitive impairment and isolated brains were later subjected to biochemical analysis to further authenticate the neuroprotective capacity of the Aa.Cr.

## 2 Methods

### 2.1 Plant extraction

The fresh leaves of *Agave americana* var. *marginata* were collected from Multan, Pakistan in the month of May, and voucher no. “R.R.Stewart 293578” was issued after authentication by an expert taxonomist. After harvesting, the leaves were cleaned to remove contamination, dried, and ground coarsely. The maceration was done in 70 % methanol in a dark color container for two weeks and then filtered. The procedure was repeated to extract the maximum of phytoconstituents (Janbaz et al., 2015). Subsequently, this collected filtrate was dried out using a rotary evaporator, and a concentrated viscous extracted material (Aa.Cr) was obtained with a yield of 12.2%, which was contained in air-tight glass bottles for subsequent experimental use.

### 2.2 Drugs and chemicals

In the present study, all used drugs and chemicals were highly purified and of analytical grade, and were dissolved freshly in normal saline before the experiment. Alloxan monohydrate and glibenclamide (Sigma-Aldrich, Germany), chloroform and methanol (Duksan pure chemicals, Korea), glucose (Merck, Germany), and normal saline (Otsuka, Pakistan) were used in the study.

### 2.3 In-Vitro experiments

#### 2.3.1 Estimation of total phenols

Aa.Cr was tested for total phenolic contents (TPC) by the Folin–Ciocalteu method and the outcomes were expressed as mg of Gallic acid equivalent per gram of extract (mg GAE/g) (Fatima et al., 2015).

#### 2.3.2 Estimation of total flavonoids

The aluminum chloride colorimetric procedure was adopted to examine the total flavonoid contents (TFC) of Aa.Cr (Fatima et al., 2015). The resultant flavonoid contents are mentioned in mg equivalents of quercetin per gram of extract (mg QE/g).

### 2.4 Antioxidant assays

The antioxidant activity of Aa.Cr was tested through the following assays: DPPH assay, phosphomolybdenum assay and potassium ferricyanide colorimetric assay, using the previously described methods (Fatima et al., 2015). A detailed methodological description of the performed experiment is given in supplementary data.

### 2.5 HPLC-DAD quantitative evaluation

For the identification of the polyphenolic compounds in Aa.Cr, a previously validated method, i.e. High-performance liquid chromatography- diode array detector (HPLC-DAD), was used (Fatima et al., 2015). The stock solutions of several phenolic standards were used and outcomes were expressed as micrograms (μg) of phytocompound per milligram (mg) of extract. The detailed methodology of the procedure is documented in the supplementary materials of the manuscript.

### 2.6 In-vivo studies

#### 2.6.1 Animals and their housing

Male Sprague-Dawley rats (150-260 g) of 4–6 weeks were procured from the National Institute of Health, Islamabad, and were accommodated in polycarbonate cages placed in the animal house of the Faculty of Pharmacy. The animals were maintained at proper conditions of temperature (25 °C), with 12 h light/dark cycle. The rats used for the study were fed with a diet enriched with high fat/carbohydrate for two weeks initially, before induction of diabetes, while regular rodent food was provided during the study with water *ad libitum*. All behavioral studies were performed from 8:00 am−6:00 pm after acclimatization of the experimental animals to the experimental conditions and environment.

#### 2.6.2 Chronic toxicity studies

Chronic toxicity of the Aa.Cr leaf extract was evaluated using a separate batch of rats with a daily administration for up to 2 months. The extract dose was increased gradually up to 1 g/kg, and animals were observed for any sign of behavioral and neurological changes. Then the optimal doses of Aa.Cr, 400 and 600 mg/kg, were selected for all activities.

#### 2.6.3 Animal grouping and respective treatments

**Fig. 1:**
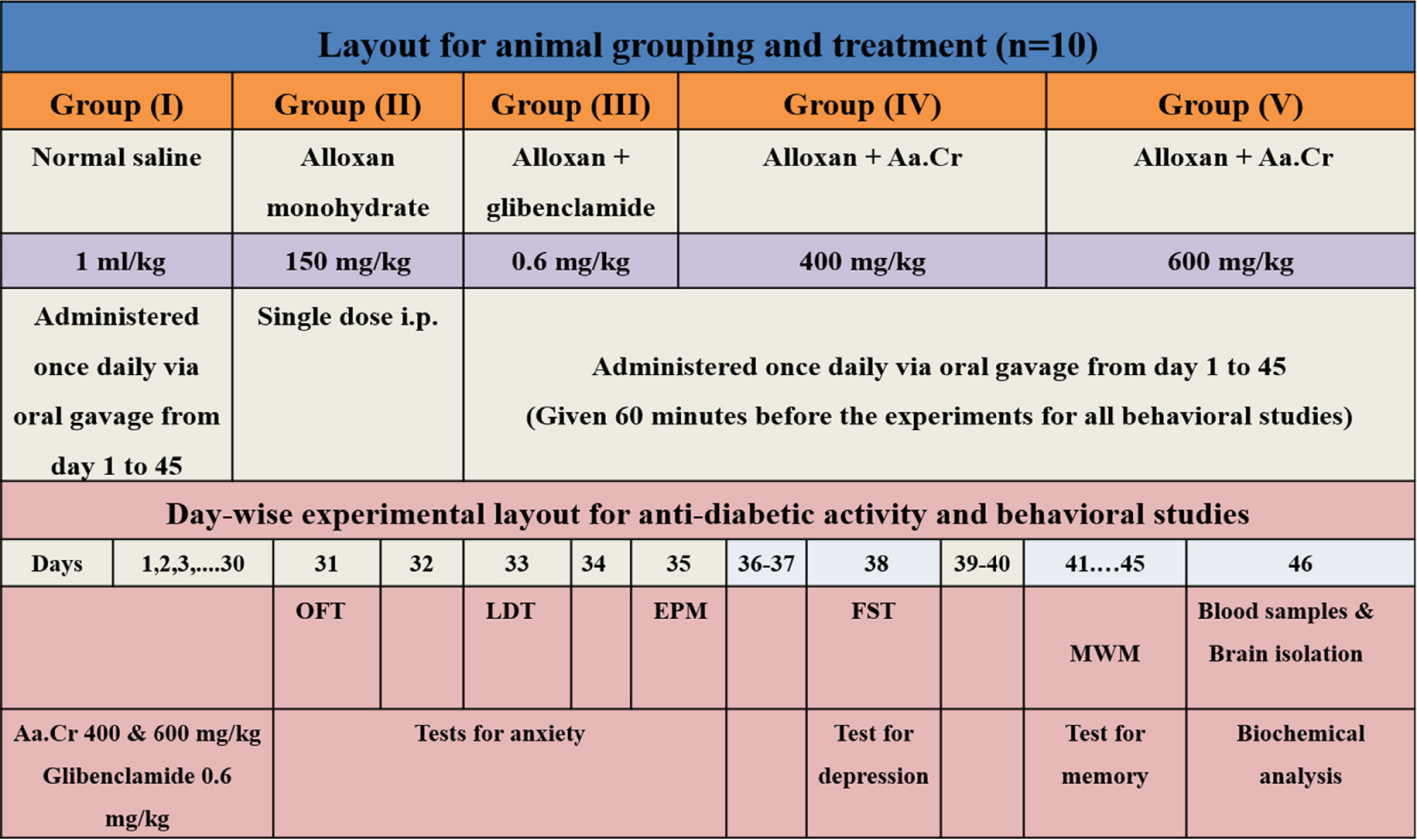
A schematic representation of the study. On day 1, the animals of groups II, III, IV, and V were injected intraperitoneally (i.p) with alloxan monohydrate (single injection). All experimental groups were subjected to behavioral experiments for anxiety on days 31–35. On day 38, the aniamls were tested for depression, followed by assessment of memory and learning from day 41 to 45. On day 46, blood and brain samples were taken for biochemical testing.

The study included five groups of randomly chosen rats (n = 10), comprising **Group I** as normal control receiving a once daily dose of normal saline (1 ml/kg). **Group II** included the diabetic control, and animals received alloxan monohydrate 150 mg/kg only (Hassanpour Fard et al., 2015, Mechchate et al., 2021). **Group III** was considered as standard and received Alloxan + Glibenclamide 0.6 mg/kg. Glibenclamide was dissolved in DMSO and further diluted in normal saline (Sedigheh et al., 2011). The animals of **Group IV** and **Group V** were administered Alloxan + Aa.Cr 400 and Alloxan + 600 mg/kg, respectively. The animals of Groups III, IV, and V received their respective treatments once daily throughout the study until the last day of the behavioral experiment, as shown in Fig. 1.

#### 2.6.4 Alloxan-induced experimental diabetes

The experimental diabetes was induced by single injection of alloxan monohydrate (150 mg/kg, i.p.) to 12 h fasted animals. Alloxan monohydrate is reported to cause apoptosis of insulin producing beta cells, which causes a sudden increase in insulin liberation in the blood, leading to a hypoglycemic shock in animals. Thus, animals were provided with 5% glucose solution orally, for the initial 2 days, in order to avoid any harmful impact of hypoglycemia (Udayakumar et al., 2009).

#### 2.6.5 Assessment of fasting glucose levels and body weights

The rats were tested for blood glucose levels using Alere glucometer test strips, by puncturing their tails after 48 h of diabetes induction. Animals with more than 260 mg/dL of fasting blood glucose (FBG) were included for further evaluation. The fasting blood glucose levels were tested every 15th day for a total of 45 days by placing a drop of tail vein blood onto the test strips of a glucometer. The animals were also monitored for changes in their body weights during the entire period of the study.

### 2.7 Behavioral studies

The animals were subjectecd to a series of behavioral studies, in order to test the impact of Aa.Cr on diabetes-associated anxiety, depression and cognitive deficits (see the detailed information of the behavioral methods in the supplementary information). On each experimental day, the animal’s acclimatization to the atmosphere of the behavioral room was done for 1 h before the experimental testing. The adminitration of respective group-wise treatments was done through oral gavage, about 0.5 h before experimentation. The experimental videos were recorded by directly positioning a Logitech camera on the apparatus, and the recording was evaluated using Any-maze software Version 05. The apparatus was intermittently cleaned with 70% isopropyl alcohol, in order to avoid the impact of any olfactory cues on the animals‘ activity during the test.

#### 2.7.1 Open field test (OFT)

To evaluate the anxiolytic potential of Aa.Cr, a square-shaped apparatus (80 × 80 cm), consisting of an acrylic material and having an open arena was used as reported previously (Imran et al., 2021, Parlar et al., 2020). The method pertaining to OFT is explained in detail in the supplementary information.

#### 2.7.2 Light and dark test (LDT)

The test equipment comprised two compartments made of plexiglass (40 × 25 × 20 cm), one light and the other dark, connected by an arc hole. It was used to check the anxiety of all the treatment groups by adopting the method previously described (Guo et al., 2013, Zhang et al., 2012).

#### 2.7.3 Elevated plus maze (EPM)

The EPM consisted of two exposed arms and two closed arms, arranged to make a plus-shaped maze (110 cm length, 10 cm width). This test was also used to reveal the anxiousness in animals of the treatment groups, using the method previously described (Javaid et al., 2021).

#### 2.7.4 Forced swim test (FST)

The FST is most widely used to assess the despair-based behavior of rodents in an unpleasant environment (Wang et al., 2017). In order to evaluate the antidepressant activity of Aa.Cr, the previously described mathod was used (Malik et al., 2020).

#### 2.7.5 Morris water maze (MWM) test

To evaluate the effect of Aa.Cr on learning and memory, rats were tested in the MWM by following the previously described method (Nurdiana et al., 2018, Sajjad Haider et al., 2021). The method pertaining to MWM is elaborated in detail in the supplementary information.

### 2.8 Biochemical parameters

#### 2.8.1 Estimation of lipid profile and hepatic marker enzymes

Blood samples were drawn by the *retro*-orbital technique as described (Patil et al., 2011) and tested for alanine aminotransferase (ALT), total cholestrol (TC), triglycerides (TG), low-density lipoprotein (LDL) and high-density lipoprotein (HDL), using Micro-lab 300 Merck kits (Eliza et al., 2009, Priscilla et al., 2015).

#### 2.8.2 Preparation of brain homogenate

On day 46, animals were arbitrarily selected from all groups (n = 4), anesthetized using 5% isoflurane and were beheaded. The levels of catalase (CAT), superoxide dismutase (SOD), glutathione peroxidase (GPx) and malondialdehyde (MDA) were estimated in the isolated brains following a previously adopted method (Asha Devi et al., 2011, Imran et al., 2021, Javaid et al., 2021).

### 2.9 Statistical evaluation

All the data were verified for normality by the Shapiro-Wilk test. Blood glucose levels, body weights, escape latencies and lipid markers were analyzed by two-way ANOVA with *post-hoc* Tukey’s test. One-way ANOVA with Tukey’s test was used to evaluate other test parameters. The data were expressed as mean ± SEM and evaluated by using GraphPad Prism (version 06).

## 3 Results

### 3.1 In-vitro experiments

#### 3.1.1 Total phenolic and flavonoid contents

The results revealed that total phenols were 51.44±16 mgGAE/g and total flavonoids were 24.95 ± 1.41 mg QE/g in the aqueous leaf extract of Agave *americana* var. *marginata* (Table1).

**Table 1:** TPC (mg GAE/g), TFC (mg QE/g), TRP (mg AAE/g), TAC (mg AAE/g), and %RSA determination in Aa.Cr.

Sample	TPC	TFC	DPPH	TRP	TAC
	mgGAE/g	mgQE/g	% scavenging	mgAAE/g	mgAAE/g
Aa.Cr	51.44 ± 16	24.95 ± 1.41	17.20 ± 1.04	228.70 ± 3.21	394.15 ± 1.34

TPC: total phenolic content, TFC: total flavonoid content, TRP: total reducing power, TAC: toal antioxidant capacity.

#### 3.1.2 Antioxidant activity

The antioxidant potential of Aa.Cr was found by discoloration of a methanolic solution of DPPH, and the IC_50_ found was 17.20 ± 1.04 μg/mL. The total antioxidant capacity (TAC) of Aa.Cr was found to be 394.15 ± 1.34 mgAAE/g in a phosphomolybdenum based assay**.** The total reducing power (TRP) of Aa.Cr was found to be 228.70 ± 3.21 mgAAE/g of the extract in the potassium ferricyanide colorimetric assay. The relative ferric reducing power of the extract from young leaves of Aa.Cr is tabulated in Table 1. Greater reducing power was indicated by elevated absorbance of the reaction mixture.

#### 3.1.3 HPLC quantitative analysis

**Fig. 2:**
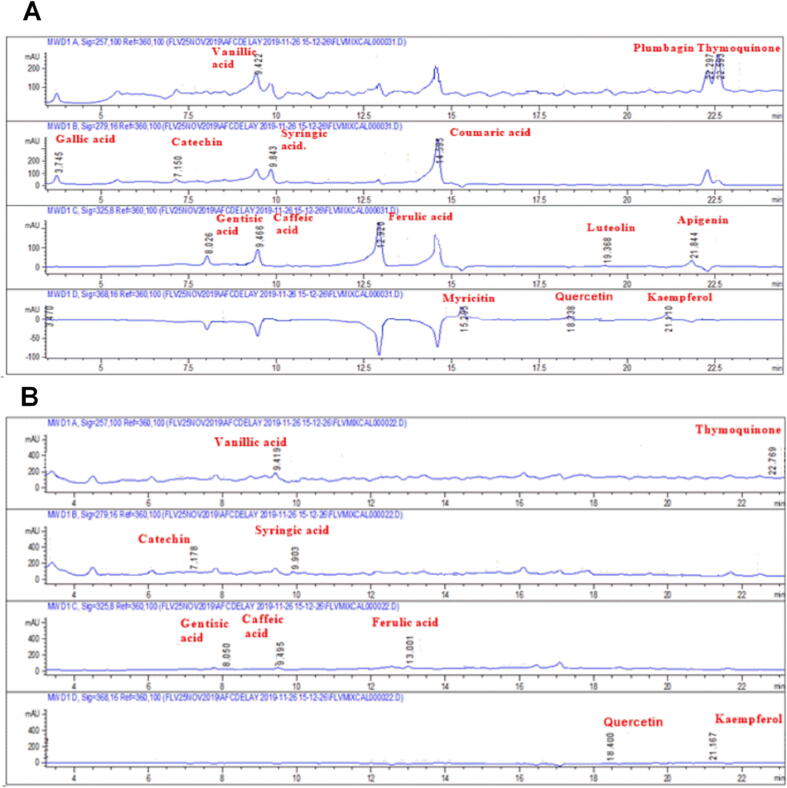
HPLC Chromatograms of flavonoid and phenolic contents, detected in the standard mixture, **(A)**, and Aa.Cr **(B)**.

The HPLC-DAD method was used to quantitatively analyze the phenols in Agave *americana* var. *marginata*. Chromatographic fingerprinting was achieved by comparison of the retention time and UV spectra of a reference compound with the test sample as given in Table 2 and Fig. 2. The leaf extract comprised 1.93 μg/mg of catechin and 1.75 μg/mg of quercetin. The occurrence of detected phytoconstituents predicts a probable potential of the plant leaves for well-known bio-activities, e.g. caffeic acid and other antioxidants.

**Table 2:** Phytoconstituents identified in the methanolic extract of Aa.Cr in HPLC analysis.

Compound	Groups	Quantity (μg/mg)
Catechin	Polyphenols	1.93
Syringic acid	Phenols	0.68
Thymoquinone	Benzoquinone derivative	0.15
Quercetin	Polyphenols	1.75
Gentisic acid	Dihydroxybenzoic acid	0.52
Caffeic acid	Hydroxycinnamic acid	0.46
Ferulic acid	Hydroxycinnamic acid	0.55
Vanillic acid	Monohydroxybenzoic acid	0.48
Kaempferol	Flavonol	0.35

### 3.2 In-vivo experiments

#### 3.2.1 Antidiabetic activity

**Fig. 3:**
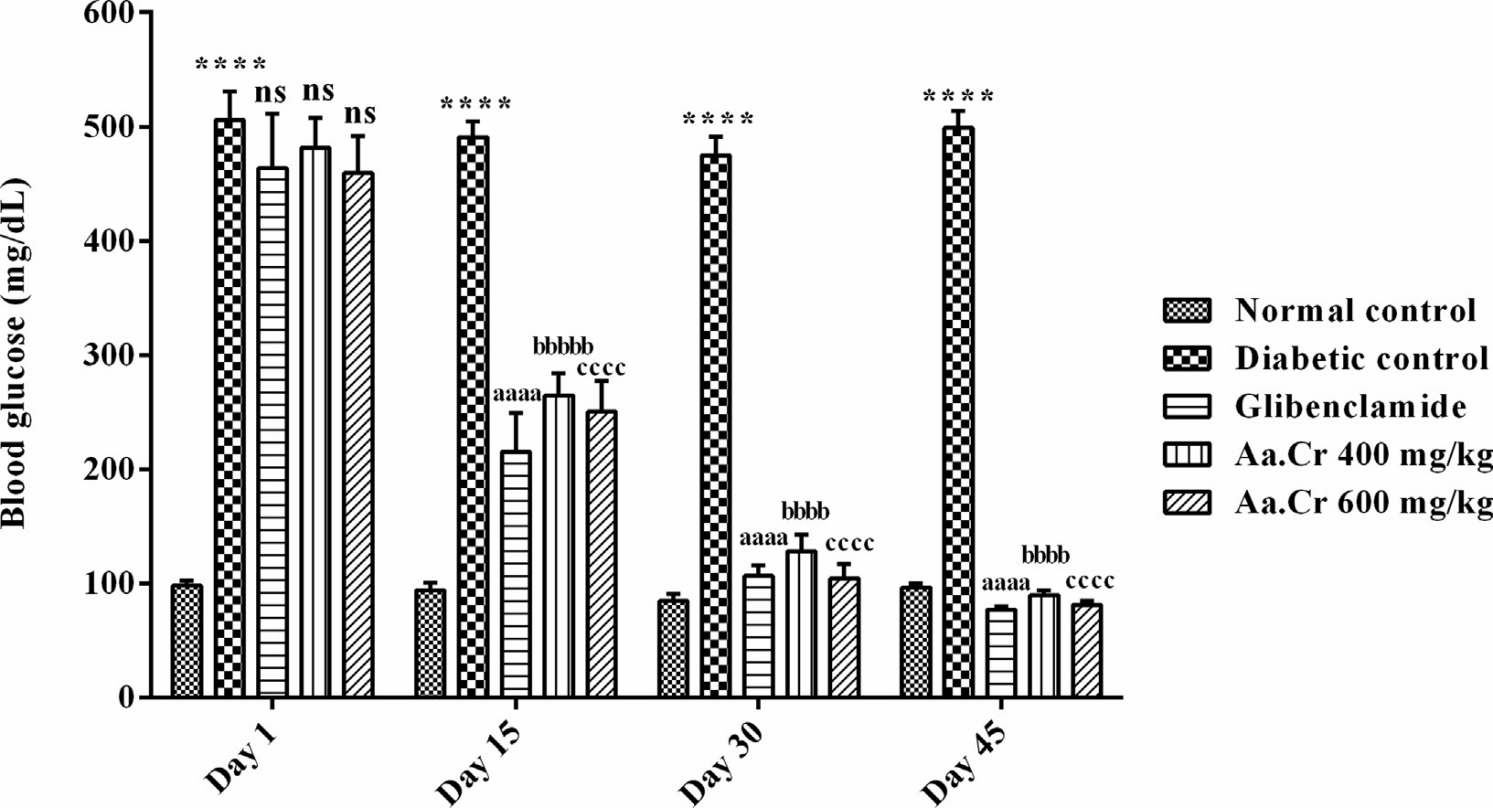
Hypoglycemic effect of chronic administration of Aa.Cr on blood glucose levels (mg/dL), monitored during 1.5 months in animals of groups I-V (n = 10). The outcomes of normal control, glibenclamide and Aa.Cr treatments were compared with the diabetic control group. *P < 0.05 and ^a^P < 0.05 show comparison of diabetic rats with normal control and the glibeclamide treated group respectively, ^b^P < 0.05 and ^c^P < 0.05 show the evaluation between diabetic control and Aa.Cr (400 and 600 mg/kg, respectively).

On day 1, the difference in blood glucose levels was not significant among the treatment groups and diabetic control groups. However, ANOVA showed a notable inter-group variation [F (4, 35) = 116.8, P < 0.0001] on the 15th day. From day 15 onwards, blood levels for glucose were increased (P < 0.0001) in diabetic rats as compared to normal control. However, these elevated blood glucose levels were remarkably reduced (P < 0.0001) with chronic treatment of rats with Aa.Cr at both doses, i.e. 400 and 600 mg/kg, in a comparable pattern as done by glibenclamide (P < 0.0001). Hence, the outcomes showed that chronically administered Aa.Cr at both doses was found to have hypoglycemic effects in diabetic rats (Fig. 3).

#### 3.2.2 Effect of the plant extract Aa.Cr on body weight

**Fig. 4:**
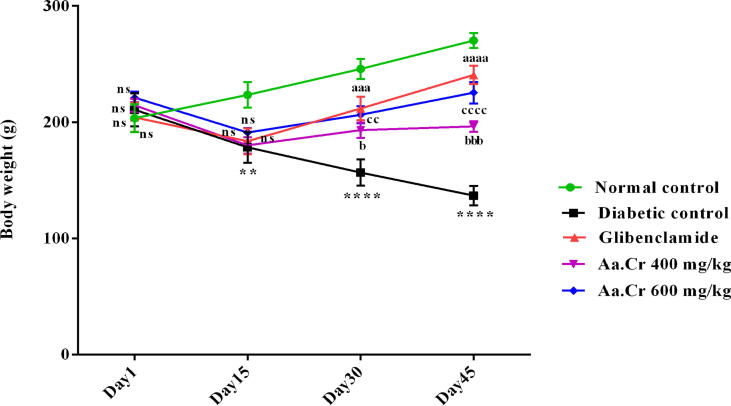
Effect of Aa.Cr on body weights (g) of animals of groups I-V (n = 10). The outcomes of normal control, glibenclamide and Aa.Cr treatments were compared with the diabetic control group. *P < 0.05 and ^a^P < 0.05 show comparisons of diabetic rats with normal control and the glibeclamide treated group, respectively. ^b^P < 0.05 and ^c^P < 0.05 show the evaluation between diabetic control and Aa.Cr 400 and 600 mg/kg, respectively.

The body weights of all groups were also measured throughout this study. After alloxan-induced diabetes, there was a considerable reduction in the weights of all the groups, especially in the diabetic untreated group, in comparison to normal control (P < 0.0001) (Fig. 4). Initially, the plant extract did not improve the body weight of animals of treatment groups from day 1 to day 15, but a gradual dose-dependent increase in body weight was observed in animals receiving chronic doses of Aa.Cr 400 (P < 0.05) and 600 mg/kg (P < 0.01) in the glibenclamide treated group (P < 0.0001), as compared to untreated diabetic animals. Furthermore, the further reduction in body weight was prominently less during 15–45 days as compared to diabetic untreated animals. The results obtained from two-way ANOVA revealed a significant inter-group variation among body weights [F (4, 140) = 26.16, P < 0.0001] and an appreciable effect of Aa.Cr at doses of 400 and 600 mg/kg (P < 0.001, P < 0.0001) to augment the bodyweight as compared to the animals of the diabetic control group who did not receive any treatment, and their weight kept on reducing day by day.

### 3.3 Behavioral studies

#### 3.3.1 Open field test (OFT)

**Fig. 5:**
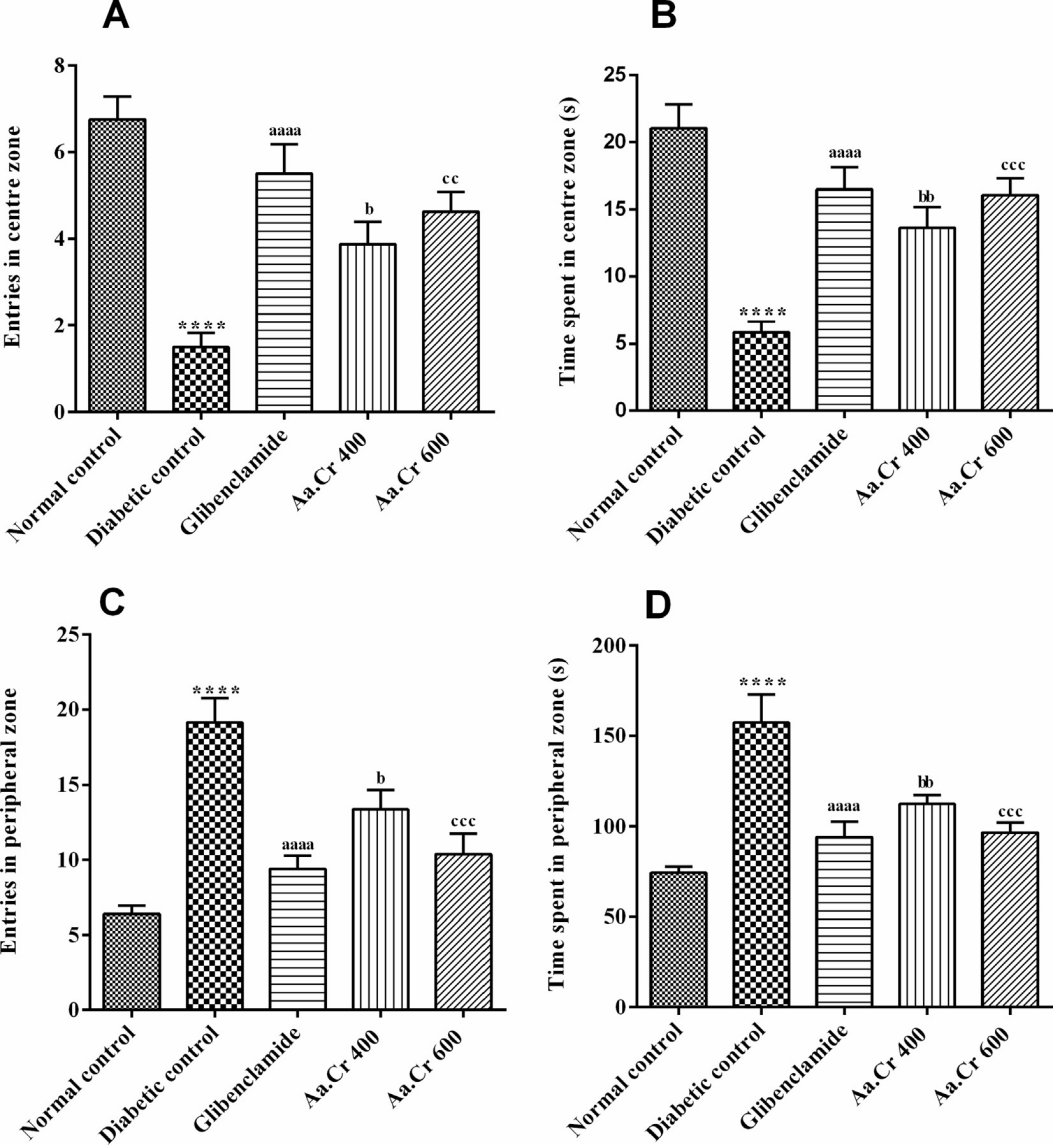
Effect of chronic administration of Aa.Cr on **(A)** entries in the centre zone, **(B)** time spent in the centre, **(C)** entries in the peripheral zone and **(D)** time spent in the peripheral zone, to evaluate the anxiety-like behavior in animals of groups I-V (n = 8) by testing in the OF test. *P < 0.05 and ^a^P < 0.05 show comparisons of diabetic rats with normal control and the glibeclamide treated group, respectively. ^b^P < 0.05 and ^c^P < 0.05 show the evaluation between diabetic control and Aa.Cr 400 and 600 mg/kg, respectively.

The animals of all groups were tested in the open field to assess the ameliorating effect of Aa.Cr on anxiety-like behavior. The animals of all treatment groups showed an increased preference for the central zone in comparison to the diabetic control group. There was significant inter-group variation for the number of entries in the centre zone [F (4, 35) = 14.57, P < 0.0001] (Fig. 5A) and the duration of stay in the centre zone [F (4, 35) = 14.73, P < 0.0001] (Fig. 5B). Hence, a significant reduction in animal’s anxiety towards the open area of the experimental apparatus was prominent in all treatment groups, as compared to diabetic untreated animals. The outcomes of one-way ANOVA with Tukey's multiple comparison test revealed that the animals treated with Aa.Cr 400 and 600 mg/kg (P < 0.01, P < 0.001) were more curious and less anxious, as shown by their increased preference for the central zone with the gradual increase in dose, as observed with glibenclamide (P < 0.0001).

Moreover, the entries in the peripheral zone and the time spent there also varied significantly between all the groups with [F (4, 35) = 15.86, P < 0.0001] and [F (4, 35) = 12.87, P < 0.0001], respectively, as depicted in Fig. 5C and Fig. 5D. The diabetic untreated animals remained in anxiety, as they had prominently lower courage to leave the peripheral area of the open field arena (P < 0.0001) as compared to the normal control group. But the animals chronically receiving Aa.Cr 400 and 600 mg/kg stayed in the peripheral zone for a shorter duration (P < 0.01, P < 0.001) like with glibenclamide (P < 0.001), as compared to the diabetic untreated rats.

#### 3.3.2 Light dark test (LDT)

**Fig. 6:**
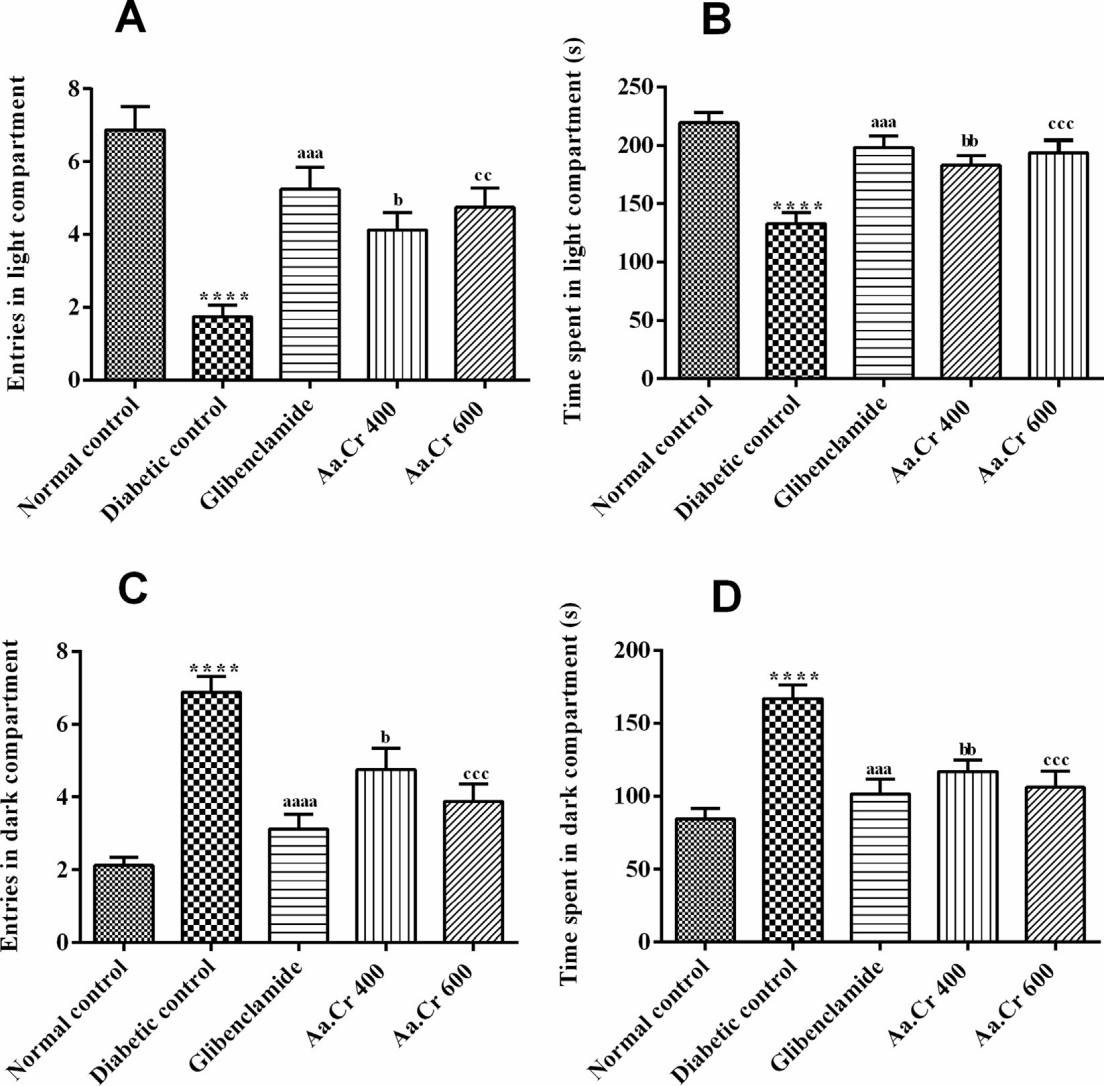
Effect of chronic administration of Aa.Cr on **(A)** entries in the light compartment, **(B)** time spent in the light compartment, **(C)** entries in the dark compartment, and **(D)** time spent in the dark compartment, to evaluate the anxiety-like behavior in animals of groups I-V (n = 8) by testing in the Light/Dark test. *P < 0.05 and ^a^P < 0.05 show comparisons of diabetic rats with normal control and the glibeclamide treated group, respectively, ^b^P < 0.05 and ^c^P < 0.05 show the evaluation between diabetic control and Aa.Cr 400 and 600 mg/kg, respectively.

LDT was performed to further check the anxiolytic potential of Aa.Cr. One-way ANOVA revealed a significant inter-group variation for the number of entries and the duration of stay in the illuminated zone of the test equipment with [F (4, 35) = 12.81, P < 0.0001] and [F (4, 35) = 11.45, P < 0.0001], respectively. In detail, the untreated diabetic rats were found reluctant towards the lightened compartment, as compared to the diabetic control group (P < 0.0001). But the Aa.Cr administration caused a dose-dependent reduction in anxiety towards the light at a dose of 400 and 600 mg/kg, as animals frequently entered (P < 0.01) and spent more duration (P < 0.001) there as compared to diabetic untreated rats (Fig. 6A and 6B).

The animals‘ choice for the innately preferred dark compartment was also monitored during this experiment. The outcomes showed a prominent variation among all groups for entries in the dark compartment [F (4, 35) = 16.56, P < 0.0001] and the duration of stay there [F (4, 35) = 11.33, P < 0.0001]. Diabetic animals were seen to reside more in the dark than control animals (P < 0.0001). However, these signs of anxiety-like behavior were noticeably reduced by Aa.Cr at 400 and 600 mg/kg (P < 0.01, P < 0.001), respectively, as shown in Fig. 6C and 6D.

#### 3.3.3 Elevated plus maze (EPM)

**Fig. 7:**
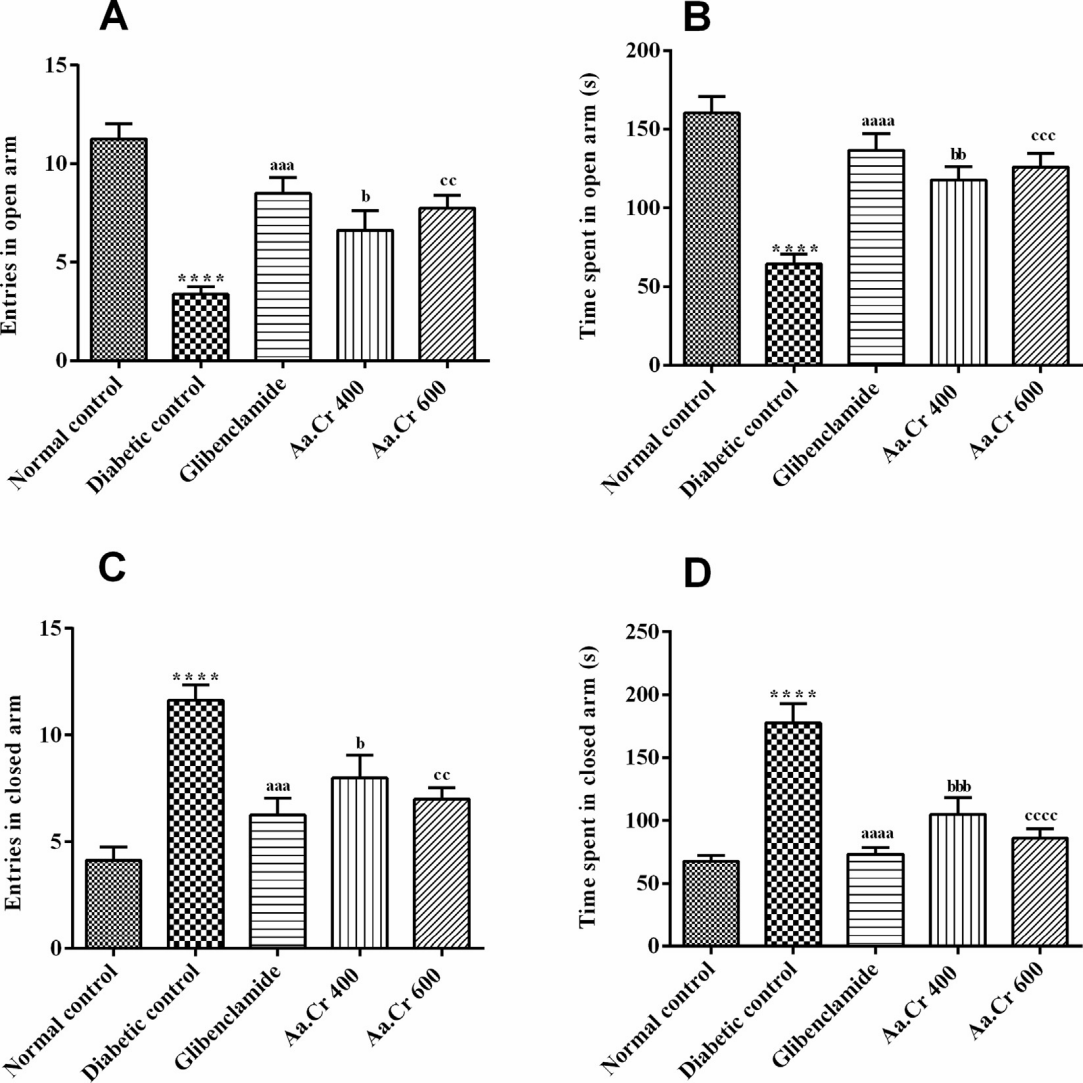
Effects of chronic administration of Aa.Cr on **(A)** open arm entries, **(B)** time spent in open arms, **(C)** closed arm entries, and **(D)** time spent in closed arms, to evaluate anxiety-like behavior in animals of groups I-V (n = 8) by testing in the EPM test. *P < 0.05 and ^a^P < 0.05 show comparisons of diabetic rats with normal control and the glibeclamide treated group, respectively, ^b^P < 0.05 and ^c^P < 0.05 show the evaluation between diabetic control and Aa.Cr 400 and 600 mg/kg, respectively.

The animals of all groups were allowed to explore the open and closed arms of the elevated plus-maze, in order to estimate their anxiousness by monitoring their choice for covered or exposed areas. The outcomes of one-way ANOVA revealed a significant difference for the number of open arm visits [F (4, 35) = 14.88, P < 0.0001] and the duration spent there [F (4, 35) = 15.72, P < 0.0001]. The animals of the normal control group were fearless as compared to diabetic rats (P < 0.0001), as they showed increased number of entries and duration of stay in the open arms of the maze. The fear seen in diabetic rats was prominently alleviated by Aa.Cr at doses of 400 and 600 mg/kg, as animals entered the open arms frequently (P < 0.01) and stayed there for a comparatively longer duration (P < 0.001), as depicted in Fig. 7A and 7B, respectively.

Likewise, a noticeable difference was observed between groups for the number of closed arms visits [F (4, 35) = 12.66, P < 0.0001] and the time spent there [F (4, 35) = 19.29, P < 0.0001]. The diabetic rats preferred the covered arms more than the normal control group (P < 0.0001), showing increased anxiety-like behavior in the diabetic group. However, this impact was reduced by Aa.Cr in a dose-dependent manner, as animals treated with 400 and 600 mg/kg showed decreased number of entries (P < 0.05, P < 0.01) and stay (P < 0.01, P < 0.0001) in closed arms of the maze, as compared to the diabetic group (Fig. 7C and 7D).

#### 3.3.4 Forced swim test (FST)

**Fig. 8:**
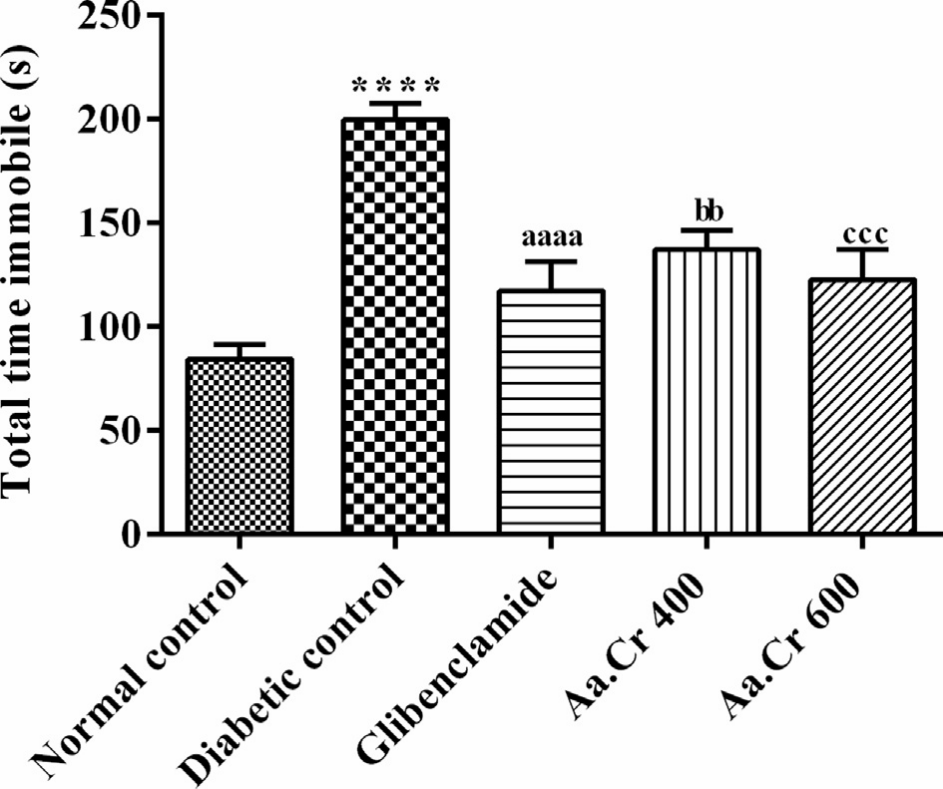
Effect of chronic administration of different doses of Aa.Cr, to evaluate depression-like behavior in animals of groups I-V (n = 8) by testing in FST. *P < 0.05 and ^a^P < 0.05 show comparisons of diabetic rats with normal control and the glibeclamide treated group, respectively. ^b^P < 0.05 and ^c^P < 0.05 show the evaluation between diabetic control and Aa.Cr 400 and 600 mg/kg, respectively.

FST was performed to assess the anti-depressant effect of Aa.Cr. One-way ANOVA described a significant variation of immobility time [F (4, 35) = 14.95, P < 0.0001] among all groups. After introducing the animals of the diabetic group to the FST apparatus, the animals remained immobile for a longer duration (P < 0.0001), showing increased hopelessness in diabetic animals in comparison to normal control. Treatment with chronic doses of Aa.Cr showed a dose-dependent reduction in this depressive behavior in rats at doses of 400 (P < 0.01) and 600 mg/kg (P < 0.001), like with glibenclamide (P < 0001). The animals were less immobile after the treatment with Aa.Cr. The immobility time of the diabetic untreated group was increased. The results are shown in Fig. 8.

#### 3.3.5 Morris water maze (MWM)

**Fig. 9:**
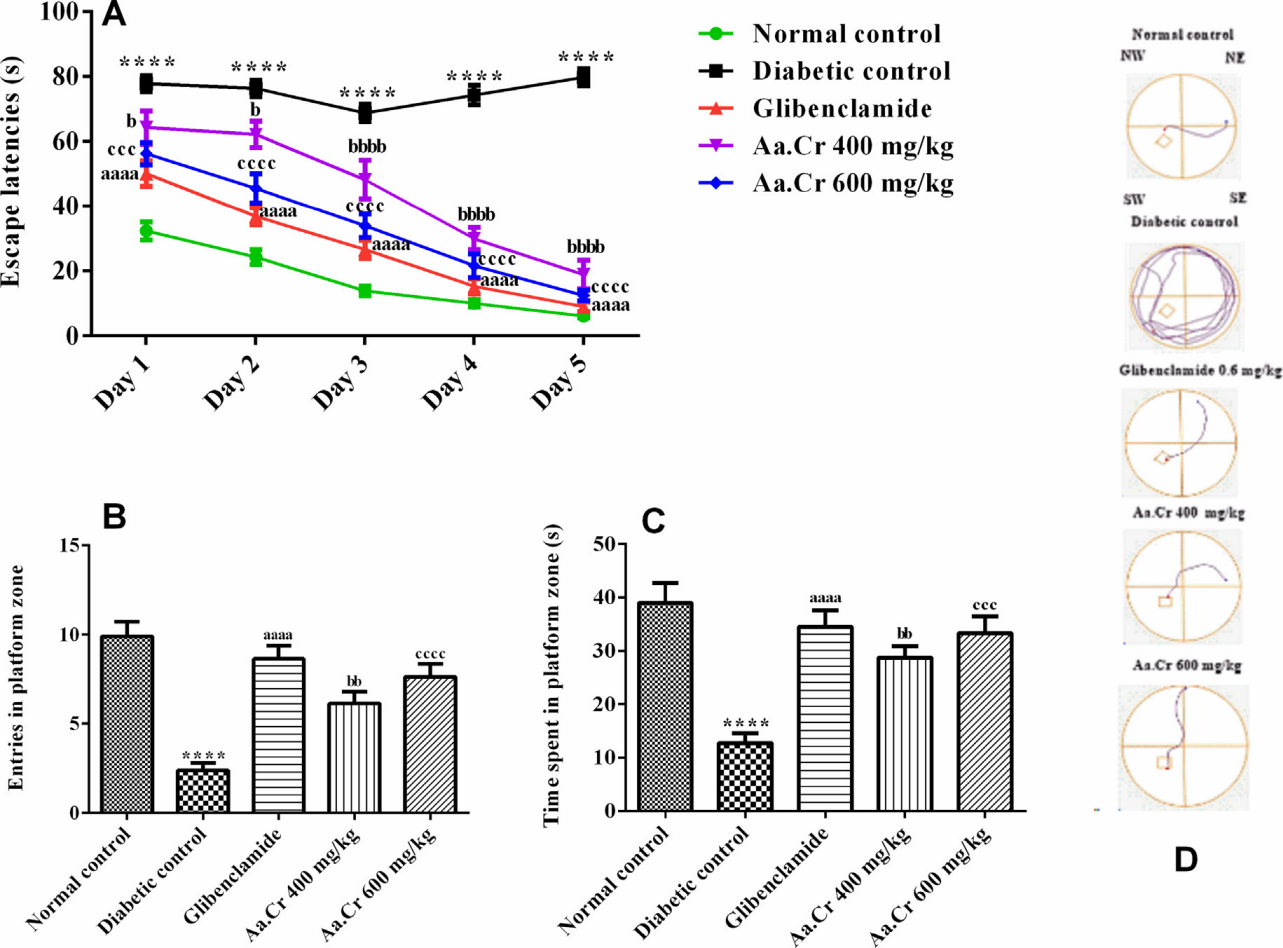
Effect of chronic administration of different doses of Aa.Cr on **(A)** escape latencies (s) during the training and experimental days, (**B)** number of entries, **(C)** time spent in the SW zone and **(D)** track plots to examine the memory difference between groups (n = 8) by testing rats in the water maze. P < 0.05 and ^a^P < 0.05 show comparisons of diabetic rats with normal control and the glibeclamide treated group, respectively. ^b^P < 0.05 and ^c^P < 0.05 show the evaluation between diabetic control and Aa.Cr 400 and 600 mg/kg, respectively.

The animals were trained for two consecutive days to locate the visible platform located in the SW quadrant of the water maze. The animals were later tested during three subsequent days for latencies to locate the submerged platform. The two-way ANOVA unveiled a significant difference for latencies to escape among all experimental groups [F (4, 165) = 248.6, P < 0.0001] during the five days (training and test). The animals of the normal control group had sufficient memory and instantly escaped to the platform, while the diabetic rats had poor remembrance of the platform because of diabetes-related dementia and showed thigmotaxic behavior and increased latencies during the five days with P < 0.0001 as compared to normal control. Diabetes-related cognitive impairment was significantly reversed (P < 0.0001) dose-dependently with chronic administration of Aa.Cr at doses of 400 and 600 mg/kg in the treatment groups, because the animals swam more quickly to find the hidden platform by recalling the position of the platform, and the latencies were decreased, the same way as with glibenclamide (P < 0.0001) (Fig. 9A).

The animals were eventually observed in the water maze without a platform on probe day. Their ability to memorize the targeted platform quadrant was checked by observing their entries and time spent in the target quadrant. One-way ANOVA showed a significant difference among all groups for the number of entries [F (4, 35) = 17.54, P < 0.0001] and duration of stay [F (4, 35) = 12.39, P < 0.0001]. In brief, the animals of the diabetic control group were moderately unable to remember the platform-stationed quadrant as they had minimum entries (P < 0.0001) and swimming duration (P < 0.0001) there, in comparison to the normal control. However, the animals which were chronically treated with different doses of Aa.Cr showed maximum entries (P < 0.0001) to the target zone where the platform had been placed during the training session, in the same manner as done by the animals treated with glibenclamide (P < 0.0001), as depicted in Fig. 9B. The swimming time of normal control and all the treatment groups in the target platform quadrant was significantly longer (P < 0.0001) as compared to the diabetic untreated group (Fig. 9C). The beneficial effect of Aa.Cr on spatial learning and reference memory in diabetic rats was noticeable at both doses of 400 and 600 mg/kg.

### 3.4 Biochemical analysis

#### 3.4.1 Anti-hyperlipidemic effects

After completion of the MWM test, blood samples were drawn to examine the effects of Aa.Cr on the lipid profiles of the experimental animals. Two-way ANOVA revealed a remarkable inter-group variation [F (4, 140) = 79.22, P < 0.0001] for the pattern of lipids in the blood samples of differently treated groups. The levels of TC, TG, and LDL were increased and HDL was significantly decreased (P < 0.0001) in the untreated diabetic control group, as compared to normal control. However, with chronic administration of Aa.Cr at doses of 400 and 600 mg/kg, these diabetes-related deteriorations were remarkably reversed. The levels of TC, TG, and LDL were decreased by Aa.Cr (400 and 600 mg/kg) with P < 0.0001, P < 0.0001 and P < 0.0001, dose-dependently. Likewise, the alloxan-induced diminished levels of good cholesterol i.e. HDL were improved by Aa.Cr (P < 0.05), like under standard antidiabetic drug treatment with glibenclamide (P < 0.01), as shown in Table 3.

**Table 3:** The levels of TC, TG, LDL, HDL and ALT in serum (n = 8), and SOD, GPx, CAT, and MDA in the brain of rats (n = 4)**.** The diabetic control group was compared to normal control, while all extract-treated groups and standard control were compared to diabetic control. One-way (ALT, SOD, GPx, CAT, MDA) and two-ANOVA (lipid profile) with Tukey's multiple comparisons test was applied, and all the obtained values are expressed as mean ± SEM; where n = 8 rats in each group. *P < 0.05 and ^a^P < 0.05 show comparisons of diabetic rats with normal control and the glibeclamide treated group, respectively. ^b^P < 0.05 and ^c^P < 0.05 are comparisons between 400 and 600 mg/kg of the extract with diabetic control.

Tests	Group I	Group II	Group III	Group IV	Group V
TC (mg/dL)	91.75 ± 4.5	212.5 ± 12.3^****^	126.12 ± 3.6**^aaaa^**	160.87 ± 7.3**^bbbb^**	144.12 ± 9.8**^cccc^**
TG (mg/dL)	76.87 ± 3.7	189.0 ± 5.5^****^	123.5 ± 6.8**^aaaa^**	157.5 ± 7.4**^bbb^**	134.37 ± 6.9**^cccc^**
LDL (mg/dL)	24.25 ± 1.3	86.37 ± 6.8^****^	38.25 ± 1.6**^aaaa^**	60.25 ± 3.3**^bbb^**	45.12 ± 3.3**^cccc^**
HDL (mg/dL)	47.25 ± 0.9	18.25 ± 1.09^**^	45.12 ± 1.0**^aa^**	40.0 ± 1.4**^b^**	42.62 ± 1.7**^c^**
ALT (IU/L)	26.63 ± 1.36	70.63 ± 5.63^****^	37.25 ± 3.15**^aaaa^**	49.47 ± 4.01**^bb^**	41.75 ± 3.87**^cccc^**
SOD (U/g)	33.75 ± 2.62	11.75 ± 1.25^****^	50.25 ± 2.52**^aaaa^**	28.25 ± 2.25**^bb^**	31.50 ± 3.01**^ccc^**
GPx (µM/min/g)	48.25 ± 2.86	30.25 ± 1.49^***^	54.25 ± 2.52**^aaaa^**	42.25 ± 2.62**^b^**	45.50 ± 2.53 **^cc^**
CAT (IU × 10^−2^)	23.50 ± 1.19	12.50 ± 0.64^***^	32.25 ± 2.42**^aaaa^**	19.75 ± 1.10**^b^**	21.75 ± 0.75 **^cc^**
MDA (µM/g)	18.75 ± 1.84	41.25 ± 3.19^***^	13.75 ± 1.93**^aaaa^**	27.50 ± 2.95**^b^**	24.25 ± 3.30 **^cc^**

#### 3.4.2 Hepatoprotective effec

The drawn blood samples were additionally subjected to evaluation of liver function using the enzyme alanine aminotransferase (ALT). One-way ANOVA showed a significant inter-group variation for levels of ALT [F (4, 35) = 18.22, P < 0.0001]. This enzyme was increased significantly in diabetic untreated animals (P < 0.0001) as compared to normal control. Fortunately, Aa.Cr ameliorated this deterioration in a dose-dependent manner, as chronic administration of Aa.Cr at doses of 400 and 600 mg/kg resulted in a significant reduction of ALT with P < 0.01 and P < 0.001, respectively, similar to glibenclamide (P < 0.0001) as shown in Table 3.

#### 3.4.3 Biochemical assessment of brain homogenate

This assay was executed to assess the levels of superoxide dismutase (SOD) in the brain of diabetic untreated rats and the treatment groups. It is an essential enzyme that controls the reactive oxygen species and increases protection against diabetes. The results obtained from one-way ANOVA showed a prominent intergroup variation [F (4, 15) = 32.61, P < 0.0001] with a significant enzyme reduction in diabetic untreated rats (P < 0.001), due to increased oxidative stress in comparison to normal control. The level of SOD was overturned dose-dependently (P < 0.01, P < 0.001) with chronic treatment of Aa.Cr at doses of 400 and 600 mg/kg, respectively, and SOD activity in the brain showed decreased oxidative stress in the same way as with glibenclamide (P < 0.0001) (Table 3).

The brains of all the groups were also further evaluated for endogenously present glutathione peroxidase (GPx) which is a protection enzyme and catalyzes the conversion of harmful peroxides to friendly products. One-way ANOVA depicted a notable difference in enzyme levels among all the groups [F (4, 15) = 13.15, P < 0.0001]. The levels of GPx were significantly reduced because of diabetes-related oxidative stress due to hyperglycemia in untreated animals as compared to the normal control group (P < 0.0001), but the oxidative stress was compensated by the antioxidant potential of GPx with chronic dosing of Aa.Cr 400 and 600 mg/kg (P < 0.05, P < 0.01), due to elevated level of GPx, like with glibenclamide (P < 0.001) (Table 3).

Catalase (CAT) is an intracellular enzyme that dissociates the hydrogen-peroxide (H_2_O_2_) to generate H_2_O and O_2_ and attenuates the level of reactive oxygen species that complement pathological conditions such as diabetes. One-way ANOVA depicted a remarkable intergroup variation [F (4, 15) = 26.61, P < 0.0001], and it was revealed that CAT activity was decreased in diabetic untreated animals due to the elevated level of oxidative stress in comparison to normal control (P < 0.0001). Interestingly, this deterioration was upturned significantly (P < 0.05, P < 0.01) with chronic administration of Aa.Cr 400 and 600 mg/kg, respectively, with a gradual increase in dose, and the plant extract was able to conserve the concentration of antioxidant enzymes and exhibited high concentrations of CAT in the brain of the treatment groups, as did glibenclamide (P < 0.0001) (Table 3).

Malondialdehyde (MDA) is an indicator of lipid-peroxidation due to oxidative stress. One-way ANOVA exposed a significant diversity in MDA levels among all groups [F (4, 15) = 14.74, P < 0.0001]. The results showed elevated MDA levels in the untreated diabetic group due to diabetes-related increased oxidative stress (P < 0.0001) as compared to normal control. Treatment with chronic administration of Aa.Cr at doses of 400 and 600 mg/kg significantly (P < 0.05, P < 0.01) reduced this lipid-peroxidation with a gradual increase in dose, like with glibenclamide (P < 0.001) (Table 3).

## 4 Discussion

The limitations of most diabetic medications in terms of poor glycemic control are widely accepted, which motivates researchers to look for novel therapeutic options to deal with this prevailing health challenge (Abbas et al., 2019, Mechchate et al., 2021). In recent decades, the plants are known as most esteemed source of novel therapeutic options as their bioactive phytocompounds exert insulin mimetic effects to counteract the hyperglycemia (Patel et al., 2012). In this study, the methanolic extract of *Agave americana* leaves exhibited a notable radical scavenging potential which might be attributed to its content of polyphenols, phenolic acids, and flavonols, detected by HPLC analysis. Flavonoids are known to have numerous health benefits against various disorders including diabetes (Sarian et al., 2017). By targeting multiple molecules involved in several pathways, flavonoids are reported to play roles in β-cell proliferation, insulin signaling and secretion (Graf et al., 2005).

The administration of alloxan resulted in hyperglycemia and a loss of body weight in rats during a period of 45 days. These parameters depicted the successful establishment of experimental diabetes in rats as the same findings were reported by Yin et al. (Yin et al., 2018). The production of reactive oxidative species increases in both types of diabetes (Matough et al., 2012). The diabetogenic impact of alloxan is precipitated by increased oxidative stress, subsequent increased cytosolic calcium and depletion of the endogenous antioxidant defense mechanisms (Ighodaro et al., 2017). The elevated oxidative stress causes oxidation of structural components of cells, thus damaging the pancreatic β-cells resulting in reduced insulin synthesis and release (Banda et al., 2018). Rats treated chronically with Aa.Cr showed a prominent hypoglycemic effect with improved body weights. These effects might be yielded through the antioxidant and anti-inflammatory potential of polyphenols and flavonoids that combat alloxan-mediated increased oxidative damage to pancreatic β-cells.

Diabetes is associated with numerous psychiatric comorbidities including anxiety, depression and intellectual deficits (McIntyre et al., 2013, Raffield et al., 2016). Buin et al. reported that diabetic people are at increased risk of developing anxiety and depression as compared to healthy individuals (Buin et al., 2020). The inclusion of behavioral experiments in the present study also revealed the presence of anxiety-like behavior in diabetic rats, as they remained in hidden and dark zones for a longer duration, but treatment with Aa.Cr provided protection against these psychological disorders. The flavonoids and polyphenols present in naturally occurring medicinal plants are reported to ameliorate anxiety-like behavior by modulating the expression of GABAergic transmission in the brain (Komaki et al., 2016, Muhasaparur Ganesan et al., 2021). Similarly, the rats treated with Aa.Cr showed dose-dependent protection from depression-like behavior, as they were more mobile in the forced swim test as compared to diabetic rats. (Hughes et al., 2016, Szebeni et al., 2017). Polyphenols might alleviate the depression by elevating the levels of monoamine neurotransmitters and brain-derived neurotrophic factor receptors (BDNF) in the brain (Li et al., 2020, Sangiovanni et al., 2017). Furthermore, the phytoconstituents with antioxidative potential might cure depressive disorders by protecting the brain from oxidative damage and related neuroinflammation. These outcomes are in accordance with Demirtaş Şahin et al. (2019) who linked the antioxidant effect exerted by chronic administration of polyphenol (resveratrol) with improvement in anxiety and depression-like behaviors in diabetic rats.

Catechin is one of the most important polyphenols possessed by Aa.Cr. Catechins have been reported to reduce anxiety and depression in an experimental rat depression model by ameliorating oxidative stress (Rai et al., 2019). A comprehensive review by Pervin et al., reports that catechins combat oxidative stress and modulate various neuronal signaling pathways and inhibit protein aggregation that collectively contribute toward their neuroprotective potential (Pervin et al., 2018). Thymoquinone is another phytochemical compound that provides antidiabetic and neuroprotective effects by modulating inflammation and oxidative stress (Abdelmeguid et al., 2010, Farkhondeh et al., 2018). In a study by Parlar et al., thymoquinone has been reported to own antioxidant and anti-inflammatory properties as its pretreatment in rats resulted in reduced malondialdehyde levels and restoration of glutathione peroxidase at site of injury (Parlar and Arslan, 2020). In another study, thymoquinone treatment resulted in reduced Inflammatory cells and pro-inflammatory and inflammatory mediators in rat model of allergic airway inflammation (Parlar and Arsalan, 2019).

The detrimental impact of diabetes on brain functionality has been widely reported in the literature (Nevo-Shenker and Shalitin, 2021, Northam et al., 2006). Though the exact mechanism is unknown, the cognitive deficit has been seen as one of the deteriorative consequences of both types of diabetes, as hyperglycemia is common (Biessels and Gispen, 2005). The alloxan-induced hyperglycemia causes increased glucose permeation in the brain leading to neuronal glucotoxicity through different mechanisms, i.e. mitochondrial dysfunction and oxidative stress (Pignalosa et al., 2021). The outcomes of the Morris water maze in this study showed that animals exposed to Aa.Cr had comparatively better remembrance of the platform as compared to diabetic rats. Polyphenols and flavonoids are broadly reported to exert neuroprotective effects by dealing with neuroinflammation and neuronal dysfunction injury and promoting the neuronal differentiation in the hippocampus (Hussain et al., 2018, Vauzour, 2012). The increased oxidative stress and lipid peroxidation in the isolated brains of diabetic rats was also noticeably improved by Aa.Cr. Polyphenols are reported to alleviate the progression of neurological disorders by increasing the expression of antioxidant enzymes and protein/cell signaling pathways (Silva and Pogačnik, 2020, Uddin et al., 2020). Thus, the antioxidant capacity of polyphenols and flavonoids might be responsible for regulating the SOD, CAT, GPx and MDA levels in rat brains (Omodanisi et al., 2017, Singh et al., 2020, Taïlé et al., 2020).

The association of metabolic disorders with atherogenic dyslipidemia has been widely reported to be due to elevated oxidative stress. Júnior et al. (2017) found an accumulation of TG, LDL, and reduced HDL levels in diabetic female rats exposed to alloxan-induced diabetes. This might be the outcome of excess mobilization of fats from adipose tissue due to the underutilization of glucose (Draganescu et al., 2021). Administration of Aa.Cr corrected the diabetes-associated hyperlipidemia in rats, thus unveiling the hypolipidemic effects of polyphenols that might act via reducing the lipid peroxidation in lipoproteins. Moreover, the polyphenols scavenge and neutralize oxidative stress, down-regulate pro-inflammatory cell signaling modulators including Nf-KB, and inhibit the arachidonic cascade and production of derivative eicosanoids (Feldman et al., 2021).

Similarly, experimental studies have previously suggested that alloxan-induced experimental diabetes can affect various organ systems including the liver (Lucchesi et al., 2015). In the present study, the increased ALT levels in diabetic rats depicted the hepatocellular injury, which might be due to the toxic action of alloxan or the animal’s diabetic state. The polyphenols and flavonoids can improve liver function by reducing inflammation and counteracting the oxidative stress in the liver as well as by modulating the insulin signaling pathways and liver gluconeogenesis (Kang et al., 2020, Yin et al., 2018).

## 5 Conclusion

In this study, the leaves of *Agave americana* var. *marginata* L. were found to be enriched with phenols and flavonoids which were capable to deal with hyperglycemia and weight reduction in alloxan-induced diabetic rats. Furthermore, diabetes-associated anxiety, depression and memory deficits in alloxan induced diabetic rats were also ameliorated by chronic administration of Aa.Cr in a dose-dependent manner. Moreover, Aa.Cr exerted protection against harmful effects of diabetes on the animals‘ liver and lipid profile. These beneficial effects might be attributed to polyphenols and flavonoids present in Agave *americana* battling with alloxan-induced oxidative stress and its deteriorative consequences. However, there is a need to explore the detailed mechanisms of the active phytoconstituents of Agave *americana* var. marginata, before employing it in future research and development.

## Declaration of Competing Interest

The authors declare that they have no known competing financial interests or personal relationships that could have appeared to influence the work reported in this paper.
