# Supplementary material for: Chronically administered Agave americana var. marginata extract ameliorates diabetes mellitus, associated behavioral comorbidities and biochemical parameters in alloxan-induced diabetic rats
Source: Saudi Pharm J. 2022 Jun 13;30(10):1373–86. doi: 10.1016/j.jsps.2022.06.003 (PMC9649343; doi:10.1016/j.jsps.2022.06.003)
Supplement: Supplementary data 1 [file mmc1.docx]

**Supplementary Information**

**Chronically Administered Agave americana var. marginata Extract in a Dose-dependent Manner Ameliorates Diabetes mellitus, Associated Behavioral Comorbidities and Biochemical Parameters in Alloxan-induced Diabetic Rats**

**Methods**

*Antioxidant Assays*

*DPPH -radical scavenging assay*

The antioxidant activity of the Aa.Cr was observed and has been evaluated by the free radical scavenging ability of 2, 2-diphenyl 1-picrylhydrazyl (DPPH) by using ascorbic acid as an affirmative control (Fatima et al., 2015). A spectro-photometric study was used to quantify the percent radical scavenging capacity (% RSA) and to find out the corresponding 50 % inhibitory scavenging concentration (SC50). The DPPH scavenging capacity was expressed as IC_50_. The four diverse dilutions of Aa.Cr (20 μL), were mixed with 180 μL of 9.2 mg/100 mL methanol DPPH solution in 96 well plates, to obtain final concentrations of 200, 66.66, 22.22, and 7.406 μg/mL. The anti-oxidant assay was performed in triplicate and the absorbance was measured at 517 nm using a microplate reader after 30 min of reaction at 37 °C. Scavenging capacity in percent (%RSA) was calculated by using the equation:

%RSA = (1-Abs/Ab_c_) × 100

Where

Abs = absorbance of DPPH solution with the sample,

Abs = absorbance of negative control containing the reagent except for the sample.

*Phosphomolybdenum assay*

The total antioxidant capacity of Aa.Cr, based on phosphomolybdenum assay, was assessed by mixing 0.1 mL of test extract (4 mg/mL DMSO) and positive control (ascorbic acid, 4 mg/mL) with 1 mL of reagent containing 0.6 M sulphuric acid, 28 mM sodium phosphate and 4 mM ammonium molybdate. A usual blank solution consisted of 1 mL of reagent solution and a suitable volume of the same solvent was used for the sample. The tubes were incubated at 95 °C in a boiling water bath for 90 min. After cooling to room temperature, the absorbance of the sample solution was measured at 695 nm against the blank using a PDA spectrophotometer (8354 Agilent Technologies, Germany). The experiment was performed in triplicate. The antioxidant activity was expressed as the number of mg equivalents of ascorbic acid per gram of dry plant weight i.e., mg AAE/g DW (Fatima et al., 2015).

*Potassium ferricyanide colorimetric assay*

The reducing power of Aa.Cr was determined according to the method described previously (Fatima et al., 2015). In short, 200 μL of Aa.Cr (4 mg/mL DMSO) was mixed with 400 μL of phosphate buffer (0.2 mol/L, pH 6.6) and 1 % potassium ferricyanide [K_3_Fe (CN)_6_]. The mixture was then incubated at 50 °C for 20 min. After incubation, trichloroacetic acid (400 μL of 10 %) was added and the reaction mixture was centrifuged at 3000 rpm for 10 minutes at room temperature. From the upper layer of solution, 500 μL was mixed with 500 μL distilled water and100 μL of FeCl_3_ (0.1 %). The absorbance of the reaction mixture was noted at 700 nm and an increased absorbance indicated increased reducing power. Blank was prepared by adding 200 μL of DMSO to the fore-mentioned reaction mixture instead of the extract. The reducing power was expressed as mg ascorbic acid equivalent per gram plant dry weight (mg AAE/g DW) and the assay was performed as triplicate analysis.

*HPLC-DAD quantitative evaluation*

High-performance liquid chromatography (HPLC) was achieved utilizing Agilent Chem- station Rev. B.02-01-SR1 (260) and Agilent 1200 series binary gradient pump coupled with diode array detector (DAD; Agilent technologies, Germany). Reverse phase chromatographic assay was performed with a Zorbex-C8 analytical column (4.6 × 250 mm, 5 μm particle size, Agilent, USA), injection volume 20 μL, and the gradient elution was conducted according to the previously described method with minor modifications (Fatima et al., 2015). The mobile phase comprised of acetonitrile-methanol–water-acetic acid in a ratio of 5:10:85:1 (solvent A) and acetonitrile-methanol-acetic acid in a ratio of 40:60:1(solvent B). The gradient method was 0–20 min for 0–50 % B, 20–25 min for 50–100 % B, and then isocratic 100 % B till 30 min. The flow rate was maintained at 1 mL/min. Stock solutions of several phenolic standards i.e., phenolic acid (gallic acid), flavonol flavonoids (quercetin, myricetin, kaempferol), flavan3-ol (catechin), hydroxycinnamate (caffeic acid),) flavonol glycoside (rutin), and flavone aglycone (apigenin) were prepared in methanol and a final concentration of 10, 20, 50, 100, 200 μg/mL was achieved after dilution. The data for peak area versus standard concentration was used to construct the calibration curve, the correlations were found to be significant at 0.05 levels, results of which are summarized in Table 2. The respective limit of detection (LOD) and limit of quantification (LOQ) as determined by linear regression analysis of the calibration curve were calculated by using the expression 3.3 * (σ/b) and 10 * (σ/b) respectively where; σ = Standard deviation of response b = Slope of the calibration curve. Before use, standard solutions, samples, and mobile phases were all degassed and filtered through a 0.45 μm membrane filter (Millipore). The absorption of Aa.Cr was recorded at 279 nm (gallic acid), 325 nm (caffeic acid), and 368 nm, (quercetin and kaempferol). The chromatographic operation was carried out at an ambient temperature and in triplicate. Before starting the next analysis, the column was serviced for 10 min and the results were expressed as mg/g DW and a comparison of retention time and UV absorption spectra of extracts with those of standards was done for the identification of compounds.

*Behavioral tests*

*Open field test (OF)*

The square-shaped apparatus (80 x 80 cm) composed of an acrylic material had an open arena surrounded by 35cm high walls to avoid animal escape. The animals were individually examined for an exploratory activity for 5 minutes in the open field. The number of entries and time spent in the central and peripheral zones were noted and the increased preferences for the central zone were taken as an indication of reduced anxiety (Imran et al., 2020).

*Light and dark test (LDT)*

The test equipment comprised of two compartments made up of plexiglass (40 x 25 x 20 cm), one light and the other dark, connected by an arc hole to allow the animal to pass through both compartments. Every animal was gently placed in the light compartment facing the wall opposite to the hole. The number of entries in the light and dark compartment and the time spent in each zone were assessed to check their anxiety behavior for 5 minutes (Guo et al., 2013; Zhang et al., 2012).

*Elevated plus maze (EPM)*

The test apparatus consisted of four arms, two exposed and two arms closed, constituting a plus-shaped maze (110 cm length, 10 cm width), and was lifted to a height of 50 cm from the ground. The open and closed arms intersected, having a central (10x10 cm centre square) platform giving access to all arms. The animals were tested individually to explore the elevated plus-maze for 5 minutes by placing the animal in the middle of the maze while facing an open arm. The time spent on the open and closed arms and the numbers of entries made into each arm were observed to reveal the anxiolytic potential of Aa.Cr as entry was defined as all four paws being positioned within one arm. Increased preference for exposed areas is considered a sign of reduced anxiety in rodents (Javaid et al., 2021).

*Forced swim test (FST)*

FST is most widely used to assess the despair-based behavior of rodents in an unpleasant environment (Wang et al., 2017). Animals were put individually into a transparent Plexiglas cylinder (35 cm height and 23 cm diameter) filled with water (25 ± 2 ^o^C) up to the level that the animal can float without touching its paws to the bottom. Each animal was tested individually for 5 minutes by releasing the animal in the test apparatus. Swimming was considered animal movement which involved at least two limbs whereas the animal was considered immobile in an upright position when any movement was absent except keeping the head above with a little float. Animal’s decreased mobility is a sign of depression-like behavior (Malik et al., 2020).

*Morris water maze (MWM) test*

To test the spatial and working memory of diabetic untreated and treatment groups, the Morris water maze test was performed (Nurdiana et al., 2018; Sajjad Haider et al., 2021).The test apparatus comprised water-filled (25 ^o^C) of a greyish, circular glass-fiber tank (150 cm diameter and 50 cm height) consisting of a square platform 10 × 10 cm. The water maze was divided into 4 poles (N, E, S, W) and 4 quadrants (NE, ES, SW, WN). The proximal and distal cues were displayed at the inner and exterior surface of the tank to assist the animals in navigation. During the initial two days, the maze comprised the visible platform placed 1 cm above the water surface. The animals were trained to locate the visible platform for 4 trials per day during these training days and if any animal could not locate the platform, then was directed to locate the platform and remained seated there for 20 seconds (s) to memorize the coordinates around the circular tank. During the subsequent 3 test days, the platform was submerged and hidden by making the water opaque with a non-toxic white dye. The position of the platform was kept constant (South-west quadrant) throughout the 5 consecutive days of the experiment. On the 6^th^ day of MWM, a probe test was carried out in which platform was taken out, and the animal was tested for 90 s to examine the reference memory by evaluating the remembrance of the target quadrant. The entries in the targeted quadrant and time spent there were recorded using a Logitech HD camera and the results were calculated using any-maze software.

*Biochemical parameters*

*Preparation of brain homogenate*

After completion of the MWM test, the animals were indiscriminately selected from all groups (n=4) and were decapitated after anesthetizing them with phenobarbital (50 mg/kg), and immediately brains were extracted on ice. Isolated brains were properly stored in normal saline at 8^°^C and for preparation of brain homogenate; 0.3 g of the brain sample (cerebellum removed) was centrifuged at 12,000 rpm with 3 mL of phosphate buffer (pH 7.4) for 20 min at low temperature. The supernatant was separated and was further assayed for biochemical investigation of the levels of superoxide dismutase (SOD), glutathione peroxidase (GPx), catalase (CAT) activity, and malondialdehyde (MDA) in the animal brain according to a previously adopted method (Asha Devi et al., 2011; Imran et al., 2020; Javaid et al., 2021).
